# Mechanism, Prevention, and Treatment of Radiation-Induced Salivary Gland Injury Related to Oxidative Stress

**DOI:** 10.3390/antiox10111666

**Published:** 2021-10-22

**Authors:** Zijing Liu, Lihua Dong, Zhuangzhuang Zheng, Shiyu Liu, Shouliang Gong, Lingbin Meng, Ying Xin, Xin Jiang

**Affiliations:** 1 Jilin Provincial Key Laboratory of Radiation Oncology & Therapy, The First Hospital of Jilin University, Changchun 130021, China; liuzj2715@mails.jlu.edu.cn (Z.L.); dlh@jlu.edu.cn (L.D.); zhengzz2715@mails.jlu.edu.cn (Z.Z.); liusy2715@mails.jlu.edu.cn (S.L.); gongsl@jlu.edu.cn (S.G.); 2 Department of Radiation Oncology, The First Hospital of Jilin University, Changchun 130021, China; 3 NHC Key Laboratory of Radiobiology, School of Public Health, Jilin University, Changchun 130021, China; 4 Department of Hematology and Medical Oncology, Moffitt Cancer Center, Tampa, FL 33612, USA; lingbin.meng@moffitt.org; 5 Key Laboratory of Pathobiology, Ministry of Education, Jilin University, Changchun 130021, China; xiny@jlu.edu.cn

**Keywords:** head and neck cancer, radiotherapy, salivary glands, injury, oxidative stress

## Abstract

Radiation therapy is a common treatment for head and neck cancers. However, because of the presence of nerve structures (brain stem, spinal cord, and brachial plexus), salivary glands (SGs), mucous membranes, and swallowing muscles in the head and neck regions, radiotherapy inevitably causes damage to these normal tissues. Among them, SG injury is a serious adverse event, and its clinical manifestations include changes in taste, difficulty chewing and swallowing, oral infections, and dental caries. These clinical symptoms seriously reduce a patient’s quality of life. Therefore, it is important to clarify the mechanism of SG injury caused by radiotherapy. Although the mechanism of radiation-induced SG injury has not yet been determined, recent studies have shown that the mechanisms of calcium signaling, microvascular injury, cellular senescence, and apoptosis are closely related to oxidative stress. In this article, we review the mechanism by which radiotherapy causes oxidative stress and damages the SGs. In addition, we discuss effective methods to prevent and treat radiation-induced SG damage.

## 1. Introduction

Head and neck cancer (HNC) is the seventh most common cancer worldwide, accounting for 3% of all cancers, with approximately 900,000 new cases and half a million deaths annually [1]. Surgery, radiotherapy, and systemic therapies (including chemotherapy, targeted therapy, and immunotherapy) play roles in varying degrees at all stages of HNC. The radiation (IR)-based approach is well-established as a standard therapy for functional preservation in patients with HNC [2].

Salivary glands (SGs) are markedly sensitive to IR therapy, and damage is generally irreversible at doses higher than 50 Gy [3]. More than 50% of patients who undergo radiotherapy involving major SGs experience the perception of hyposalivation, termed IR-induced xerostomia [4]. Xerostomia is one of the most detrimental long-term side effects of multimodal therapy in patients with locally advanced HNC [5]. The clinical manifestations of xerostomia include dysgeusia, dysphagia, painful swallowing, and difficulty sleeping and speaking, which are closely related to the patient’s quality of life [6].

Radiotherapy significantly increases reactive oxygen species (ROS) levels, thereby inducing oxidative stress in SGs [7]. Oxidative stress plays an important role in the calcium signaling of SG and microvascular damage. In addition, a large number of trials have been conducted to treat IR-induced SG injury (RISGI). In this review, we discuss the mechanism of IR-induced SG damage through oxidative stress, summarize how to improve the secretory function of SGs, and propose some suggestions for future research.

## 2. Mechanism of RISGI

Bergoni first described the phenomenon of RISGI. Oral and pharynx tissues exposed to IR have short- and long-term sequelae, including the loss of SG function and persistent xerostomia [8]. These problems are caused by damage to highly radiosensitive acinar SG cells (fluid-producing) [9]. Initially, based on the slow turnover rate of SG cells (60–120 days), it is expected that the SGs will be unresponsive (>60 days). However, the changes in saliva production and composition shortly after radiotherapy indicate that these glands respond quickly [10,11]. Many early morphological studies on the effect of radiotherapy on salivary tissues have shown that serous cells are more sensitive than mucous cells. Therefore, the parotid gland is more likely to cause damage during radiotherapy in HNC patients [9,12,13]. Moreover, recent clinical studies on parotid and submandibular/sublingual saliva flow after radiotherapy have shown that the dose effect is similar in saliva composition and flow rate changes [10,11,14,15].

In the first few days after radiotherapy, parotid gland function may be reduced by 50% [16]. The earliest hypothesis, specifically the degranulation theory, was that IR damage caused the loss of salivary acinar cells. The hypothesis of degranulation is that radiotherapy damages the peroxidation of the redox-capable iron and copper ions on cell membrane lipids, leading to the leakage of proteolytic enzymes from the secreted granules and triggering cell lysis. Later, it was discovered that the SGs consist of a variety of cells with different metal and enzyme contents. This evidence does not support the degranulation hypothesis. Clinical studies have also confirmed that the stimulation of pilocarpine before irradiation leads to the degranulation of the parotid glands, which produces only 13% protection [17]. In irradiated cells, the behavior of particles is not different from that of unirradiated cells. Therefore, we rejected the degranulation hypothesis.

Although the catheter loses its function, most of its structure remains intact [18]. In the acute phase, IR mainly suppresses water secretion and causes a sharp drop in saliva flow [19].

## 3. Calcium Signaling

Under physiological conditions, acetylcholine acts on G-protein-coupled receptors. The G-protein-coupled receptor contains seven transmembrane alpha helices, which are involved in the process of signal transduction. The G-protein-coupled receptor that binds to the ligand undergoes a conformational change. By exchanging guanosine diphosphate originally bound to G protein with guanosine triphosphate (GTP), G protein separates the α subunit from the β and γ subunits. This process activates the G protein (specifically the α subunit bound to GTP). The activated G protein then activates the downstream phospholipase C (PLC). After that, phosphatidylinositol 4,5-bisphosphate is decomposed into inositol triphosphate (IP3) and diacylglycerol (DAG) under the action of PLC and activates the G-protein effector PLC by activating the G protein; thus, phosphatidylinositol 4,5-bisphosphate is decomposed into IP3 and DAG. Among them, the DAG–protein kinase C alpha (PKC) pathway is related to the secretion of SG proteins. IP3 acts on the IP3 receptor on the endoplasmic reticulum, intracellular Ca^2+^ is released, and the intracytoplasmic Ca^2+^ concentration increases. As a second messenger, Ca^2+^ increases intracellular Ca^2+^ ([Ca^2+^i]) through Ca^2+^ sensor protein stromal interaction molecule 1 (STIM1), which regulates the storage operation Ca^2+^ entry (SOCE). This process is controlled by channel transient receptor potential criterion 1 and [Ca^2+^i]-release-activated calcium channel protein 1 [20,21,22,23,24]. Elevated [Ca^2+^i] regulates the activity of ion channels, generating an appropriate osmotic gradient to drive fluid secretion across the apical membrane.

Initially, it was generally believed that the intracellular Ca^2+^ concentration and the muscarinic receptor-mediated Ca^2+^ mobilization state in irradiated cells are equivalent to those of unirradiated cells, and the degree and time course of Ca^2+^ mobilization are not affected by IR therapy. However, intracellular Ca^2+^ depletion did not explain the decline in cell proliferation [25,26].

Subsequent research showed that when exposed to a muscarinic acetylcholine receptor agonist, the ability of irradiated SG cells isolated from the body to mobilize [Ca^2+^i] after irradiation is closely related to the oxidative stress caused by IR. IR causes nicotinamide adenine dinucleotide (NAD+) and ROS to accumulate in damaged tissues. External NAD^+^ may be converted to adenosine diphosphate ribose (ADPR), cADPR, and nicotinic acid adenine dinucleotide phosphate by the ectoenzymes CD38 and CD157. Extracellular ADPR may then bind to plasma membrane receptors, increase [Ca^2+^i] through the release of Ca^2+^ from stores via G proteins, and activate PLC with subsequent IP3 production. H_2_O_2_ (a type of ROS), may also cross the plasma membrane and mobilize ADPR from mitochondria (both H_2_O_2_ and cADPR can synergize with ADPR to activate transient receptor potential cation channel, subfamily M, member 2 (TRPM2)). TRPM2 is an ROS-sensitive non-selective channel with a unique C-terminal ADPR pyrophosphatase domain [27,28]. Free cytosolic ADPR acts on the plasma membrane TRPM2 channels, enabling Ca^2+^ influx across the plasma membrane and/or the release of lysosomal Ca^2+^, thereby increasing the Ca^2+^ concentration in the cytosol [29,30].

TRPM2-mediated [Ca^2+^i] increases trigger mitochondrial Ca^2+^ uniporter-dependent Ca^2+^ uptake into mitochondria. As a result, [Ca^2+^] and ROS levels in the mitochondria increase while the mitochondrial membrane potential decreases. In addition, the slow emergence of activated caspase-3 eventually leads to the loss of STIM1. The loss of STIM1 leads to a decrease in SOCE, which, in turn, decreases saliva secretion. This phenomenon is observed in the acinar cells of the submandibular glands of TRPM2^+/+^ mice in the early stage after irradiation, but it does not occur in the acinar cells of TRPM2^−/−^ mice [30].

**Figure 1:**
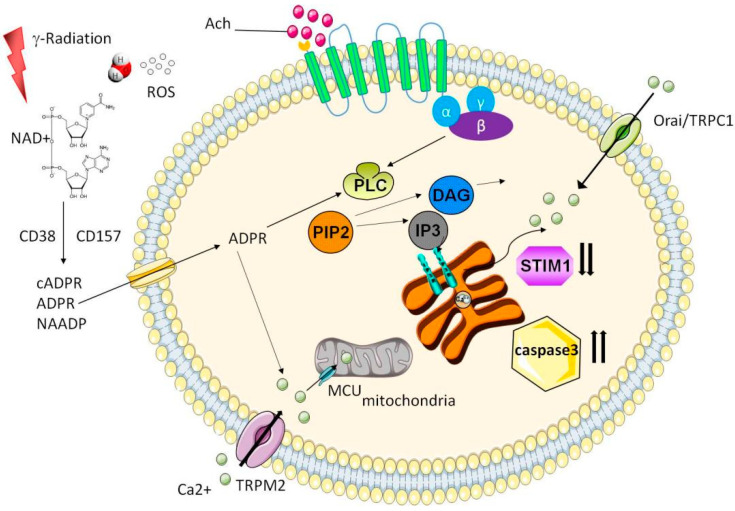
Calcium signal mechanism of radiation-induced salivary gland injury. Acetylcholine acts on G-protein-coupled receptors and activates the G-protein effector PLC by activating the G protein so that PIP2 is decomposed into IP3 and DAG. IP3 acts on the IP3 receptor on the endoplasmic reticulum, and then intracellular Ca^2+^ is released and the intracytoplasmic Ca^2+^ concentration increases. As the second messenger, Ca^2+^ increases [Ca^2+^i] through Ca^2+^ sensor protein STIM1, which regulates the SOCE. This process is controlled by the channels TRPC1 and Orai1. When exposed to a muscarinic acetylcholine receptor agonist, the irradiated salivary gland cells isolated from the body are impaired in their ability to mobilize [Ca^2+^i] after irradiation. NAD+ and ROS accumulate during inflammation and tissue damage. External NAD+ may be converted to ADPR, cADPR, and nicotinic acid adenine dinucleotide phosphate by the ectoenzymes CD38 and CD157. Extracellular ADPR may then bind to plasma membrane receptors, increase [Ca^2+^i] through the release of Ca^2+^ from stores via G-proteins, and activate PLC with subsequent IP3 production. H_2_O_2_ may also cross the plasma membrane and mobilize ADPR from mitochondria (both H_2_O_2_ and cADPR can synergize with ADPR to activate TRPM2). Free cytosolic ADPR will act on the plasma membrane TRPM2 channels, enabling Ca^2+^ influx across the plasma membrane and/or the release of lysosomal Ca^2+^.

In addition to the mechanism in which changes in [Ca^2+^i] are mediated by TRPM2 reduces saliva secretion, the cell-membrane-binding disorder of PKC has also been confirmed in previous studies. Phosphorbol esters (PMA), analogs of diacylglycerol, can activate PKC. When the SG cells isolated after irradiation are exposed to muscarinic acetylcholine receptor agonists, the muscarinic acetylcholine agonist-induced activation of PKCα is severely affected in irradiated cells in vivo. Similarly, PMA is no longer able to activate PKCα in irradiated cells. In addition, IR can interfere with the binding of PKCα to the membrane, resulting in impaired intracellular signal transduction. This study showed for the first time that the ability of cells to respond to receptor agonists is impaired after IR [31]. The calcium signaling mechanism of RISGI is summarized in Figure 1.

## 4. Microvascular Injury

Microvascular damage may play an important role in RISGI [32]. Exposure to 15 Gy induced a 50% reduction in microvascular density (MVD) within the SGs of C3H mice [33]. IR-induced microvascular dysfunction that leads to a decrease in MVD in SGs results from oxidative stress caused by IR, and the role of ROS is crucial [34]. Studies using bovine aortic endothelial cells have shown that ROS induces extensive apoptosis in a short period of time. The oxidative stress response is achieved through membrane lipid peroxidation. Acid sphingomyelinase hydrolyzes sphingomyelin in the plasma membrane of endothelial cells to produce ceramide. Ceramide, acting as a second messenger in receptor-mediated signal transduction, initiates endothelial cell apoptosis through the mitochondrial system [35]. IR-induced DNA damage can also initiate ceramide generation via the activation of mitochondrial ceramide synthase and the de novo synthesis of ceramide. BAX is activated downstream of ceramide, regulating vascular endothelial cell apoptosis via the release of mitochondrial cytochrome c [36,37].

## 5. Decreased Parasympathetic Nerve Signals

Under physiological conditions, the nerve growth factor secreted by epithelial cells acts on the parasympathetic nerves to maintain the function of parasympathetic neurons. Acetylcholine secreted by the parasympathetic nerve acts on the cholinergic receptors muscarinic 1 (Chrm1) and Chrm3 of SOX2+ SG stem cells. Acetylcholine acts on Chrm1 to activate the transactivation of the heparin-binding epidermal growth factor (HB-EGF) pathway, which promotes the proliferation and differentiation of progenitor cell epithelial cells. The activation of the HB-EGF pathway is related to the expression of genes related to cell cycle progression, such as cyclin A2 [38,39]. SOX2 is a marker of progenitor cells in the submandibular and sublingual SGs of fetal mice, and it is essential for the regeneration of salivary acinar cells [40].

Radiotherapy can reduce the occurrence of epithelium and cause the loss of epithelial terminal buds. The apoptosis of epithelial cells reduces the expression of brain-derived neurotrophic factor (BDNF), neurotrophic factor, and nerve growth factor, which directly causes the downregulation of parasympathetic nerve function. The downregulation of Chrm1 and Chrm3 transcription levels and the decline of stem cells’ ability to respond to acetylcholine reduce the number of stem cells [41,42,43]. Radiotherapy additionally reduces the expression of neuronal tubulin (Tubb3), which is a marker of axons in parasympathetic nerves and has previously been shown to affect the maintenance of epithelial progenitor cells [38].

## 6. Water Channel Hypothesis

The decrease in saliva flow after radiotherapy is disproportionate to the loss of acinar cells. The gland volume does not change after irradiation; only the salivation function is impaired. This indicates that RISGI may be the cause of low salivary function [18]. Aquaporin (AQP) is a constitutively activated water channel that does not exhibit a polarized membrane distribution in most epithelia [44,45]. AQP1 and AQP5 play important roles in the hypofunction of SGs caused by IR. AQP1 is a constitutively activated water channel that does not show a polarized membrane distribution in most epithelial cells [44]. Furthermore, AQP1 is only detected in the venules and capillaries within SGs [46]. AQP5 is mainly located on the apical membrane of the secretory acinar cells of the rat submandibular, parotid, and sublingual glands, as well as on the apical membrane of the ductal cells inserted into the rat submandibular gland [47]. A number of studies have used mice, rats, and humans to increase the expression of human AQP1 (hAQP1) in the SG parenchyma by constructing a recombinant adenovirus encoding hAQP1 [48,49,50]. The salivary flow rate of SGs after gene transfer significantly increases. At the same time, studies have shown that the degree of improvement in SG function is inconsistent. Subsequent studies found that the label density of AQP1 and Na^+^/K^+^-ATPase did not significantly decrease after irradiation, and only the AQP5 protein was significantly lost [51,52]. These studies emphasized the important role of AQP5 in the reduction of SG flow caused by IR and explained the inconsistent improvement in SG function after AQP1 gene transfer [49,53]. The level of AQP5 protein is regulated by the parasympathetic nerve/M3 muscarinic receptor agonist. Cutting the parasympathetic nerve can reduce the amount of AQP5 protein. This process is achieved by the degradation of AQP5 by autophagosomes and/or lysosomes [54,55]. IR can reduce the function of parasympathetic nerves. Therefore, we infer that IR can indirectly reduce the level of the AQP5 protein by inhibiting the function of the parasympathetic nerves. The mechanism underlying the reduction in AQP1 protein levels is still unclear.

## 7. Cellular Senescence and Apoptosis

Cell senescence is a stable state of cell proliferation, and the loss of SG function after IR is closely related to this mechanism. The levels of ROS and oxidative stress malondialdehyde in the irradiated SGs were significantly increased. This process is achieved through the p16 INK4a/Rb-dependent pathway induced by growth/differentiation factor-15. ROS accelerate the shortening of telomeres through oxidative stress and directly damage DNA, thereby inducing cell senescence. DNA damage is a sign of cell senescence. When oxidative stress causes DNA damage in SG cells, ataxia telangiectasia mutation kinase is the first to respond to DNA double-strand breaks (DSBs), and its substrates include histone H2AX and p53 binding protein-1 (53BP1). Phosphorylated H2AX and 53BP1 are quickly localized in DSBs, forming characteristic lesions in the nucleus. In addition, IR can cause the expression of senescence-related markers in SG cells (senescence-related β-galactosidase (SA-βgal), p19ARF, and DcR2) and increase the secretion of senescence-related phenotypic factors (PAI-1 and interleukin 6 (IL-6)), which indicates the occurrence of cellular senescence. Among them, IL-6-mediated cellular senescence plays an important role in SG damage caused by IR, and IL-6 signal transduction depends on the expression of IL-6R and its co-receptor gp130 on target cells. When IL-6R is not expressed, IL-6 can also signal by acting on the soluble receptor. This mechanism is called IL-6 trans-signaling [56]. IL-6R is mainly expressed in ductal cells. Using IL-6 knockout mice, experiments have shown that IR-induced DNA damage induces cell senescence and hypofunction through the continuous expression of IL-6 in the SGs [7,57]. In contrast, the characteristics of cellular senescence, such as mitochondrial dysfunction and p21 upregulation, increase ROS production, which may help stabilize senescence [58].

In addition to cell senescence, cell apoptosis is a possible cause of RISGI. Experiments on rhesus monkeys have shown that irradiated serous SGs undergo interphase cell death due to apoptosis [13]. The main reason for the massive loss of acinar cells in animal models after IR has caused widespread controversy. First, it was discovered that the necessary condition for IR to cause the apoptosis of SGs was the expression of p53 in the body. P53 is a tumor suppressor that is closely related to the regulation of DNA damage repair, cell cycle arrest, and cell apoptosis [59].

There are complicated mechanisms by which IR activates p53 in salivary glands through oxidative stress. Among them, the nuclear translocation of PKCδ is essential for inducing apoptosis [60]. PKCδ is a member of the lipid-regulated serine/threonine PKC family. At the same time, it is also an important regulator of mitochondrial-dependent apoptosis in epithelial cells. Studies have shown that IR strongly induces apoptosis in wild-type mouse parotid gland cells in vivo, while PKCδ−/− mouse parotid gland cell apoptosis is inhibited by more than 60% [61]. In addition, the reintroduction of PKCδ through adenovirus transduction can restore the apoptotic response. Before the cell is exposed to pro-apoptotic factors, the N-terminal regulatory domain of PKCδ restricts the nuclear localization sequence (NLS)-dependent PKCδ input into the nucleus. PKCδ exists in the cytoplasm in a conformation that excludes importin-α binding. When PKCδ is in an inactive state, the intramolecular contact between C2 and the catalytic domain prevents the NLS from binding to importin-α [62].

Tyr-64 and Tyr-155 are the main sites of tyrosine phosphorylation in response to H_2_O_2_ [63]. When H_2_O_2_ is produced by the oxidative stress of IR, phosphorylation at PKCδ-Y64 and Y155 opens the PKCδ conformation. Exposure to NLS allows both Hsp90 and importin-α to bind to the binding sites of PKCδ. PKCδ accumulates in the nucleus during the binding of importin-α [62]. This step is necessary to induce apoptosis; however, the phosphorylation of PKCδ is not necessary for apoptosis, because in the physiological state, after the addition of SV-40 nuclear localization signal, the non-cleavable form of PKC delta also enters the nucleus to induce cell apoptosis [64].

When phosphorylated PKCδ enters the nucleus, PKCδ can control the expression of p53 in a transcription-dependent manner in the nucleus, and it can also activate p53 via the phosphorylation of different serine and threonine residues in an indirect manner. After IR causes DNA damage, PKCδ activates the Bcl-2-related transcription factor and binds it to the CPE region of p53, thereby inducing p53 transcription [65]. PKCδ can also be activated by IKKα caused by oxidative stress and indirectly activates p53 through the phosphorylation of ser20 [66]. The phosphorylation of p53 allows for the stabilization and activation of p53 to promote gene expression, thereby inducing cell cycle arrest and apoptosis. Activated p53 can, in turn, promote PKCδ transcription in response to DNA damage [67].

In addition, PKCδ entering the nucleus can also be cleaved by nuclear caspase-3 to produce proapoptotic δCF [68]. The expression of δCF is associated with the activation of the pro-apoptotic protein Bax and cytochrome c release [36]. In addition, δCF further activates caspase-3 in the cytoplasm through positive feedback [69].

**Figure 2:**
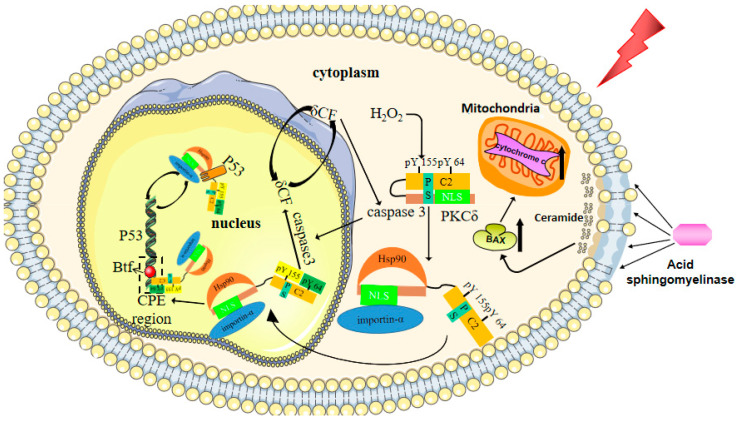
**The mechanism of cell apoptosis in radiotherapy-induced salivary gland injury**. When H_2_O_2_ is produced by the oxidative stress of radiation, phosphorylation at PKCδ-Y64 and Y155 opens the PKCδ conformation. The exposure of NLS allows both Hsp90 and importin-α to bind to the binding sites of PKCδ. Under the binding of importin-α, PKCδ accumulates in the nucleus. When phosphorylated PKCδ enters the nucleus, PKCδ can control the expression of p53 in a transcription-dependent manner in the nucleus and activate p53 via the phosphorylation of different serine and threonine residues in an indirect manner. After radiation causes DNA damage, PKCδ activates Bcl-2-related transcription factor (Btf) and binds with it to the CPE region of p53, thereby inducing p53 transcription. At the same time, activated p53 can in turn promote PKCδ transcription in response to DNA damage. PKCδ entering the nucleus can also be cleaved by nuclear capsase-3 to produce pro-apoptotic δCF. Acid sphingomyelinase hydrolyzes sphingomyelin in the plasma membrane of endothelial cells to produce ceramide. Ceramide, acting as a second messenger in receptor-mediated signal transduction, activates BAX. The elevation of BAX regulates vascular endothelial cell apoptosis by releasing mitochondrial cytochrome c.

In short, both cell senescence and apoptosis are the mechanisms by which radiotherapy causes a loss of SG function. IL-6 plays an important role in inducing cell senescence. For apoptosis, oxidative stress products produced by IR act on PKCδ, which activates and increases p53 transcription in the nucleus through nuclear translocation and can also activate cell apoptosis through interaction with caspase-3. The cell senescence mechanism of RISGI is summarized in Figure 2.

## 8. Treatment of RISGI

### 8.1. Amifostine

RISGI is closely related to free radicals produced by radiotherapy. As a free radical scavenger, amifostine can effectively reduce the SG damage caused by radiation [70,71]. At first, amifostine is effective in reducing oral problems caused by salivary dysfunction after radiotherapy (tooth caries, oral candidiasis, and mucositis) [72,73,74]. Subsequent clinical studies and meta-analyses have shown that the application of amifostine together with radiotherapy for HNC can improve xerostomia. The incidence of long-term xerostomia was found to be lower in amifostine groups [75]. In addition to reducing the severity of xerostomia, the use of amifostine during head and neck radiotherapy can reduce the duration of xerostomia after 2 years [76].

Amifostine is effectively concentrated in saliva tissues and is activated as a selective tissue protective metabolite. Compared with normal cells, the relative lack of amifostine in tumors indicates that it can protect normal tissues and increase the radiotherapy index [77]. The use of amifostine does not reduce the local control rate, progression-free survival, and overall survival [76,77,78,79]. Intravenous injection of amifostine has a good saliva preservation function without tumor protection [80,81]. Subcutaneous treatment appears to be equally effective as a method of administration [82]. Recently, a study on the subcutaneous injection of amifostine showed that the serum concentration of the active metabolite is comparable to that of intravenous injection and the clinical benefit in reducing xerostomia is also similar [83]. On this basis, its side effects may be less than that of intravenous injection. The subcutaneous injection of amifostine is more convenient and safer [84]. 

However, some people have questioned the effect of amifostine in preventing xerostomia [85]. Although there is a statistical difference in the improvement of xerostomia after the application of amifostine, the advantage is not obvious [79]. At the same time, intensive radiotherapy and chemotherapy regimens also cause the incidence of xerostomia and mucositis [86].

### 8.2. Antioxidant Stress Therapy

Radiation produces a lot of reactive oxygen species. These reactive oxygen species, especially the excessive formation of the superoxide (SO) free radical O_2_, play a central role in the occurrence and development of xerostomia and mucositis. The natural antioxidant enzyme superoxide dismutase (SOD) in the human body detoxifies superoxide by converting it into hydrogen peroxide (H_2_O_2_), a relatively stable and less reactive oxidant. However, during radiotherapy, the excessive production of superoxide free radicals overwhelms the natural SOD enzyme and leads to SO-mediated damage, which makes it possible for exogenous superoxide dismutase and antioxidants to reduce the toxicity of radiotherapy.

Cationic Mn (III) N-substituted pyridylporphyrins (MnPs) are powerful SOD mimics. It was found that they can quickly react with many other species and change the activity of transcription factors, such as nuclear factor kappa B, hypoxia-inducible factor-1α, nuclear factor E2-related factor 2 and specific protein 1 [87,88,89,90]. Among them, MnTnBuOE-2-PyP^5+^ (BMX-001) and Mn (II) pentaaza macrocycle (GC4419) play important roles in the prevention and treatment of the oral mucositis and salivary gland damage caused by radiotherapy. In addition, it is surprising that both of these two kinds of Mn porphyrins can exert anti-tumor effects in combination with radiotherapy.

GC4419, a highly stable low molecular weight MnPAM dismutase mimic, is a selective superoxide dismutase mimic that has the characteristics of high cell permeability, high stability, and high selectivity to O_2_ [91]. M40403 (GC4403) is the enantiomer of GC4419. Previously, researchers used mice to obtain convincing results in the prevention and treatment of radiation oral mucositis [92]. In 2013, GC4419 was introduced into clinical trials to verify its role in reducing the normal tissue toxicity caused by radiotherapy. Like M40403, GC4419 can effectively prevent and reduce radiation-induced normal tissue toxicity in a mouse model of radiation-induced oral mucositis [93]. The efficacy of GC4419 in reducing the oral mucositis caused by radiotherapy and chemotherapy in patients with oral or oropharyngeal cancer was confirmed in a phase 1b/2a trial, and its safety is tolerable [94]. A subsequent phase IIb clinical study showed that 90 mg of GC4419 significantly reduced the duration, incidence, and severity of severe oral mucositis, as well as having acceptable safety [95]. A phase III trial (ROMAN; ClinicalTrials.gov identifier: NCT03689712) has begun. In addition to protecting normal tissues, avasopasem manganese also has the effect of sensitizing tumor cells to radiation-induced damage [96]. GC4419 combined with hyperfractionated radiotherapy can inhibit tumors in non-small cell lung cancer, pancreatic ductal adenocarcinoma, and head and neck squamous cell lines. Research using a radiation-resistant SqCC/Y1 HNSCC cell line and high-dose radiation (12 Gy per fraction) combined with GC4419 enhanced the response of tumors in SqCC/Y1 [91]. GC4419 increased the level of Bax in the lung tissue induced by IR, decreased the level of Bcl-2, and increased the ratio of Bax/Bcl-2. GC4419 increases TrxR activity in normal cells but reduces the activity in lung cancer cells, thereby increasing the sensitivity of cancer cells to oxidative stress. This mechanism needs to be confirmed in head and neck tumors [96].

BMX-001 is a potent manganese porphyrin SOD mimic and a regulator of cellular redox signaling pathways. Studies have used C57BL/6 mice to prove that this substance can reduce the incidence of mucositis and xerostomia after receiving local radiotherapy in the head and neck. What is particularly surprising is that BMX-001 significantly increases the number of M1 macrophages in tumors, thereby producing an inhibitory effect on tumor tissues. The FaDu human epithelial cell line was used for this study [97]. Another study showed that BMX-001 had a radioprotective effect on normal tissues at both the early and late time points. Although they did not confirm the tumor suppressor effect of BMX-001, at least they confirmed that drug does not reduce the efficacy of radiation and cisplatin, which may be related to the dose of MnP [98]. A trial of BMX-001 treatment combined with radiotherapy and cisplatin for locally advanced head and neck cancer is currently underway (NCT02990468).

The nitroxide Tempol is an SOD mimic and effective antioxidant [99]. Tempol and other nitroxide analogs have been shown to prevent radiation-induced damage in in vitro and in vivo models [100,101]. In a mouse model, Tempol has been shown to be effective against IR-induced salivary gland damage and subsequent reduction in saliva flow [102,103] and IR-induced oral mucositis [104]. A clinical trial is currently underway to assess the ability of Tempol to prevent and/or reduce the toxicity associated with cisplatin and radiotherapy in patients with head and neck cancer (NCT03480971). Epigallocatechin-3-gallate (EGCG) is a well-known antioxidant. It is the main flavonoid produced during the heating of Camellia sinensis (green tea) leaves [105]. As early as 2004, a study using human immortalized salivary acinar and ductal cells confirmed that EGCG can protect normal salivary gland cells from damage induced by chemotherapy or radiotherapy [106]. A recent study conducted a comprehensive study of the biological effects of EGCG in the pro-acinar and ductal epithelial compartments of the intact SG organ. SG pretreated with 7.5 µg/mL of EGCG promoted epithelial proliferation and the development of pro-acinar buds and ducts in regular homeostasis. In addition, EGCG’s antioxidant activity after IR injury effectively prevents epithelial cell apoptosis [107]. As a strong antioxidant, α-lipoic acid (ALA) is highly reactive to free radicals that can prevent normal tissue damage and dysfunction caused by radiation by increasing the level of glutathione in the tissues; this has been confirmed in the small intestine and thyroid of rats [108,109]. ALA in the salivary glands can also effectively improve weight loss and decrease the salivary secretion caused by radiation. The mechanism is able to inhibit the apoptosis and senescence of acinar cells and ductal cells, as well as inhibit the reduction of AQP-5 expression in salivary gland epithelial cells [110,111]. In addition to the abovementioned antioxidants, thymoquinone, vitamins, melatonin, resveratrol, and other substances have shown good antioxidant activity in the salivary glands of irradiated rats and mice [112,113,114,115]. The antioxidant effect of vitamins was confirmed in a prospective randomized controlled trial [116]. However, the antioxidant effects of thymoquinone, melatonin, and resveratrol in preventing radiation-induced salivary gland damage in the human body still need to be confirmed by further clinical studies.

### 8.3. Growth Factor Therapy

Growth factors, such as insulin growth factor 1 (IGF-1), epidermal growth factor (EGF), and fibroblast growth factor (FGF), have the ability to repair RISGI. In order to protect the vascular endothelium, FGF activates the FGFR–PI3K signal, thereby protecting the SGs from IR-induced apoptosis and maintaining acinar structure and function [117,118,119]. There have been many studies on the protective effects of IGF on SGs. IGF-1 promotes DNA repair in the parotid gland through the maintenance and activation of sirtuin-1 and can restore SG function by normalizing cell proliferation and improving the expression of amylase [16,120]. EGF can protect salivary gland function by supplementing KRT14+ ductal progenitor cells and MIST1+ acinar cells. In addition, EGF has an anti-apoptotic effect, which is achieved by activating the EGFR–PI3K–AKT pathway [121]. Heat shock protein-25 (HSP25) and HSP70i have similar effects. HSP25 or HSP70i transfer inhibits IR-induced apoptosis in acinar cells, granular coiled cells, and intercalated ductal cells. In addition, it has an opposite effect on the expression of AQP5 in SGs inhibited by IR. However, the transfer of HSP25 reduces the content of amylase, protein, Ca^2+^, Cl^−^, and Na^+^ in saliva. IGF-1 can also increase the binding of p21 and p53 by reducing the binding of ΔNp63 to the p21 promoter after IR. Since the use of IGF-1 after radiotherapy can increase the transcription of p21, it can increase the accumulation of cells in the G2/M phase, inhibit cell apoptosis, and restore SG function [122]. Similar to the role of IGF-1, roscovitine, an inhibitor of the cell cycle, acts to transiently arrest the cell cycle at the G2/M phase by competing for the ATP binding site in the catalytic cleft of the cyclin-dependent kinase. Roscovitine-induced transient G2/M cell cycle arrest can effectively inhibit cell apoptosis, thereby maintaining normal salivary function after the targeted IR of the head and neck [123]. On the basis of confirmation that IGF-1 can improve the hypofunction of SGs caused by radiotherapy, although IGF-1 administered at the same time as radiotherapy leads to an increase in the tumor cell growth rate compared to the use of IGF-1 after radiotherapy, IGF-1 is effective in radiotherapy for head and neck tumors. The efficacy of these cells has no significant impact [124]. Recently, IGF-1 has been shown to restore salivary function by maintaining the activation of atypical protein kinase C and partially rescuing the cellular positioning of YAP in label-retaining cells. This mechanism is necessary for IGF-1 to restore SG function. The specific mechanism of action remains to be explored [125].

### 8.4. Molecular Targeted Therapy

#### 8.4.1. Targeted TGF-β Therapy

The protective effect of simvastatin on SGs may be attributed to the elimination of malondialdehyde, the reduction of collagen deposition, and the reduction and delay of IR-induced increase in the expression of transforming growth factor β1 (TGF-β1) [126]. Hyperbaric oxygen therapy (HBOT) can also inhibit TGF-β, which is related to IR-induced fibrosis and chronic functional loss after IR therapy. In addition, HBOT reduces the expression of fibrosis-related factor α-smooth muscle actin in the SGs, enhances the proliferation of the SGs, and increases the vascular density of the SGs [127].

#### 8.4.2. Targeted PKCδ Therapy

Tyr-64 and Tyr-155 tyrosine phosphorylation can activate PKCδ by promoting nuclear import in response to apoptotic stimuli. C-Abl was identified as PKCδTyr-155 kinase, and c-Src was identified as a Tyr-64 kinase. Dasatinib can block the phosphorylation of PKCδ at Tyr-64 and Tyr-155. Imatinib specifically blocks PKCδTyr-155 phosphorylation. Both block the combination of PKCδ and importin-α and nuclear input to inhibit the nuclear accumulation of PKCδ. Moreover, dasatinib can inhibit etoposide, and IR can induce apoptosis in vitro [63]. The continuous administration of dasatinib can extend the protection period to at least 5 months, and the combined use of dasatinib or imatinib with fractional IR does not enhance tumor growth [128]. In addition, TKIs mediate radioprotection by increasing the repair of DNA double-stranded breaks, and TKIs increase the IR-induced activation of DNA-PK but not ATM. Moreover, TKIs increase the basal and IR-induced expression of genes associated with NHEJ (DNA ligase-4, Artemis, and XLF) and HR (Rad50, Rad51, and BRCA1). TKIs provide radioprotection to the salivary gland tissues and support the clinical exploration of TKIs in head and neck cancer patients undergoing IR therapy [129].

#### 8.4.3. Targeting PI3K/AKT/mTOR Pathway

The mammalian target of rapamycin (mTOR), a highly conserved serine/threonine protein kinase, is responsible for cell growth, protein synthesis, metabolic homeostasis, and cancer progression. The administration of the mTOR inhibitor rapamycin was found to significantly prevent the IR-induced decline of saliva in miniature pigs and to protect human SG cells from cell depletion and the loss of proliferation after IR. This protective effect is accompanied by a decrease in DNA damage and the upregulation of mitochondrial superoxide dismutase [130] (Table 1).

### 8.5. Stem Cell Therapy

#### 8.5.1. Save SCs to Reduce IR Damage

SG recovery depends on the exposure dose and stem cell (SC) level. Salivary stem/progenitor cells (SSPC) are present in the SG ducts of mice. Tissue remodeling has been achieved through SC expansion, and its effect has been confirmed in limited experiments in mice and humans [131]. SC-based therapies can provide ways to alleviate the decrease in radioactive salivation in patients after radiotherapy [132]. Deferoxamine can prevent IR-induced SG dysfunction in mice by preserving more SCs. The mechanism may be to increase hypoxia-inducible factor 1α to reduce acinar cell apoptosis and increase the expression level of VEGF to increase angiogenesis in damaged tissues [133]. The role of adipose-derived SCs in restoring SG function after IR is also related to angiogenesis, and they help restore blood flow in submandibular gland tissue.

Lin^−^CD24^+^c-Kit^+^Sca1^+^ SCs have the highest proliferation, self-renewal, and differentiation abilities during continuous passage in vitro. Studies have found that glial cell-derived neurotrophic factor (GDNF) is highly expressed in Lin^−^CD24^+^c-Kit^+^Sca1^+^ SCs. The expression of GDNF in Lin^−^CD24^+^c-Kit^+^Sca1^+^ SCs was found to be upregulated in the submandibular glands of mice and humans after radiotherapy. GDNF enriched the number of functional acinars in the irradiated submandibular glands and enhanced the salivary formation of salivary SCs but did not promote the growth of HNC cells. This process may be achieved through the GDNF–RET signaling pathway [134]. Interestingly, radiotherapy itself can also cause the upregulation of GDNF expression in Lin^−^CD24^+^c-Kit^+^Sca1^+^ SCs of the submandibular gland in mice and humans. The regulation of the GDNF pathway has a bright future for the treatment of RISGI [135].

Now, more and more evidence shows that the Wnt/β-catenin signaling pathway is essential for maintaining and activating different types of SCs. The central mediator of Wnt signaling is β-catenin, which is an activator of gene transcription. SGSCs have been confirmed to exist in SG ducts. The concurrent transient activation of the Wnt pathway can prevent chronic SG dysfunction after IR by acting on SG duct EpCAM(^+^) cells, inhibiting apoptosis, and preserving functional salivary stem/progenitor cells [136,137]. However, it is worth noting that male mice were found to improve SG function through Wnt/β-catenin signal activation, but no signal activation was observed in female mice. Wnt/β-catenin signal activation may lead to the increased radioresistance of cancer stem cells [138]. Therefore, the mechanism of activating the Wnt/β-catenin pathway to protect SGs from radiotherapy still needs to be confirmed by further clinical prospective experiments. 

Studies have investigated the improvement of SG function by injecting SCs into the SGs of IR-treated mice and humans, and it has been found that the therapeutic effect is good, which may be related to the normalization of blood vessels and the reduction of fibrosis [139,140]. In addition, experiments have compared the ability of young and old SCs to improve SGs according to the activity of SCs at different ages. The results showed that although the number of aging SGSCs was reduced, there was no difference between young SGSCs in vitro [141].

Bone marrow cell extracts play a significant role in restoring the normal function of SGs. Granulocyte colony stimulating factor (G-CSF) or FMS-like tyrosine kinase-3 ligand, stem cell factor, and G-CSF (called F/S/G) combine to mobilize BMC after radiotherapy, promote the regeneration of submandibular glands, reduce SG vascular damage, and directly promote the formation of new blood vessels [142]. Bone marrow cell extracts of both fresh and bone marrow cells can restore saliva secretion; increase cell proliferation; upregulate regeneration/repair genes; and protect salivary acinar cells, parasympathetic nerves, and blood vessels from the SGs damaged by IR [143,144]. Cell extracts from whole bone marrow, spleen, and adipose-derived stromal cells can restore 65–70% of saliva secretion; protect acinar cells, blood vessels, and parasympathetic nerves; and promote cell proliferation [145]. For the best timing and frequency of using bone marrow cell extracts, some test results have shown that starting the injection of bone marrow (BM) soup within 1–3 weeks can reduce the impact of IR-induced SG damage and improve the recovery of saliva function. Although the therapeutic effect of BM soup weaken after 8 weeks, it can be maintained by increasing the frequency of weekly injections [146].

#### 8.5.2. Other Functioning SCs

Side population cells have been identified as putative SCs in various organs. Recent in vitro and in vivo studies have shown that although the transplanted side population cells are only sparsely distributed and do not produce any growth products, they can use cluster proteins to inhibit oxidative stress and oxidative stress-induced cell damage to restore SG function after IR. Clusterin is essential for this process [147]. In recent years, research on mesenchymal SCs has increased daily. Bone marrow mesenchymal cells derived from bone marrow and adipose tissue act on SSPC by secreting growth factors to proliferate and improve dry mouth. Trials have used mesenchymal stem cells (MSCs) to treat patients with Sjogren’s syndrome, and it has been found that they are successful in maintaining the salivary function of non-obese diabetic mice; the cells positive for AQP5, AQP4, α-SMA, CK5, and c-Kit are retained, the gene expression of AQP5, EGF, FGF2, BMP7, LYZ1, and IL-10 is upregulated, and the gene expression of TNF-α, TGF-β1, MMP2, CASP3, and IL-1β is downregulated [148]. The level of serum EGF and proliferation rate of the glands also increased. The specific mechanism by which growth factors improve SG function is described below. Studies have shown that mice transplanted with BM-cMSCs have a significant increase in salivary flow rate 12 weeks after transplantation. The management of BM-cMSC was found to retain the microscopic morphology of the SG, and the SGs transplanted with BM-cMSC showed more functional recovery than the SGs injected with PBS (control group) [149]. Therefore, this method can be used to improve IR-induced advanced fibrosis in SGs. When SG damage is expected to be low, the technique using bone marrow-derived cells is sufficient to limit xerostomia after radiotherapy; therefore, this therapy depends on the number and function of the remaining cells. When the damage is expected to be high, the bone marrow-derived cells are insufficiently mobilized, so SC transplantation is required [150] (Table 2).

### 8.6. Gene Transfer Therapy

#### Transferred Genes

Radiotherapy can reduce the expression of AQP on the surface of salivary gland acinar cells. Experiments have been conducted using adeno-associated virus type 2 (AAV2) to deliver hAQP1 to the parotid glands of piglets and mice with damaged radiotherapy, causing the surviving duct cells to secrete more fluid [151,152,153]. This helps improve saliva function in patients who have previously received IR therapy. 

Previous experiments have been conducted in animals. The results of a test of delivering the AdhAQP1 vector to a single human parotid gland showed that this process is safe; the transfer of hAQP1 cDNA increased parotid gland flow and relieved the symptoms of some subjects [50]. Zheng et al. further stated that the human cytomegalovirus enhancer/promoter (hCMVp) in AdhAQP1 was probably not methylated in transduced human SG cells of responding subjects, resulting in an unexpectedly longer functional expression of hAQP1 [154]. When researchers transduced mouse and rat cell lines in vitro and submandibular glands in vivo with AdhAQP1, the methylation time of hCMVp was found to be longer. This phenomenon can explain the decrease in the expression and function of hAQP1 in vitro and hAQP1 expression in vivo [154]. 

Sonic hedgehog (Shh) gene transfer inhibits IR-induced cellular senescence by promoting DNA repair and reducing oxidative stress, which is mediated by the upregulation of the expression of genes related to DNA repair (such as survivin and miR-21) and inhibits the expression of the pre-senescence gene Gdf15, which is downstream of miR [7]. In addition, Shh gene transfection can activate HedgehogGli signaling. The transient activation of the Hedgehog pathway after IR reduces SG function in male rats by preserving salivary stem progenitor cells, parasympathetic innervation, and capillaries [155]. A previous study showed that IR can significantly reduce vascular endothelial growth factor A (VEGFA) mRNA expression, while Ad–Shh but not Ad–GFP transfer significantly retained VEGFA expression in the 4th week, and Shh gene transfection could also activate autophagy and inhibit fibrogenesis in illuminated glands [155]. SG resident macrophages are essential for functional SG regeneration following IR, which is related to their paracrine interactions with endothelial and epithelial cells. Hedgehog activation after IR recovers SG resident macrophages, either directly or by inducing the expression of Csf1 and IL34 in epithelial progenitors and endothelial cells, which then restores the expression of macrophage-derived Hgf and C1q [156]. C1q also enhances phagocytosis by tagging damage-associated molecular patterns and apoptotic cells, and it promotes the polarization of macrophages to the M2 phenotype and the production of anti-inflammatory cytokines [157,158].

The preservation of parasympathetic nerves is related to the expression of neurotrophic factors, such as BDNF and neurturin (NRTN). Improving microvascular function may be achieved by inducing angiogenic factors [131,159]. NRTN is mainly secreted by epithelial cells and essential for parasympathetic neuron survival and axon growth. Mice with the genetic deletion of either NRTN or its receptor Gfrα2 have fewer parasympathetic neurons, as well as reduced SG innervation and function. The AAV2 vector expressing human neurotrophic factor (CERE-120) has been used to treat mouse mandibular glands before or after IR. Pre-IR (not post-IR) treatment with CERE-120 can prevent functional decline. CERE-120 prevents the hypofunction caused by IR, restores immune homeostasis, and has coordinated responses to the damage or treatment of the contralateral glands. In porcine PGs, CERE-120 gene therapy has been found to improve salivary function and to reduce IR-induced alterations in acinar structure to control levels 16 weeks after IR. Treatment with CERE-120 in pig PGs can also reduce IR-related immune responses and fibrosis, as well as improve secretion [50,160]. CERE-120 gene therapy is a potential treatment for head and neck cancer patients to influence communication among neuronal, immune, and epithelial cells to prevent IR-induced salivary hypofunction and restore immune homeostasis. In addition to neurotrophic factors, the upregulation of nerve growth factor in human SG cells and mouse submandibular gland tissues mediated by AAV can also effectively reduce IR-induced human SG cell apoptosis. This process is achieved by the dephosphorylation of JNK kinase. It is not achieved by dephosphorylating JNK kinase instead of promoting AKT phosphorylation [161]. Researchers previously conducted a phase I human clinical trial in which the adenovirus gene AQP1 was transferred to a single SG of xerostomia caused by IR. However, due to its short cycle and heavier immune response, it cannot be widely used in phase II trials [162]. Since then, studies have tried to mediate more stable gene transfer in animal parotid glands through the serotype 2 AAV2 vector, and the effect has been good [163]. According to recent findings, as a means to provide AQP1 gene therapy in IR pig models, urinary angiotensinogen can replace adenovirus vectors and is a candidate for phase I human clinical trials [164].

In summary, gene transfer technology brings hope for the restoration of SG cell function after radiotherapy. The hAQP1 gene was found to effectively increase the salivary flow of the parotid glands in both animals and humans. Shh gene transfer can also preserve SG function by preserving SG stem progenitor cells, parasympathetic nerves, and capillaries. Genes expressing human neurotrophic factors can prevent decreased salivary secretion by reducing the immune response, fibrosis, and restoring neuronal function (Table 3).

## 9. Conclusions

The mechanism of RISGI is closely related to oxidative stress. ROS generated by IR increase the concentration of calcium ions in the cytoplasm by cooperating with ADPR to activate TRPM2 channels. The activation of Ca^2+^ uniporters results in changes in the osmotic gradient of Ca^2+^. In addition to the Ca^2+^ signal, microvascular endothelial cells undergo apoptosis, and the microvessel density decreases. IR can also cause a decrease in the parasympathetic nerve function that innervates the SGSCs and decreases the response of stem cells to acetylcholine. Cell senescence and apoptosis are two important factors in the loss of function of SG cells caused by IR. IR accelerates the shortening of telomeres through oxidative stress and directly damages DNA, thereby inducing cell senescence.

Growth factors can protect SG function by inhibiting IR-induced apoptosis. SCs are important for the long-lasting secretion of SGs. The choice of gene transfer vector is controversial.

Based on current evidence, the mechanism of RISGI is complex and diverse. Several treatments have been explored. In addition to traditional surgery, pilocarpine, amifostine, and submandibular gland surgical transfer, new treatment methods for SCs have emerged in recent years. In the future, antioxidant drugs based on SOD mimics and gene transfer research based on gene expression may improve the quality of life of clinical patients.

## Figures and Tables

**Table 1:** Drug treatment of radiation-induced SG injury.

Treatment	Radiation Dose	Medicine	Drug Dosage	Mode of Administration	Routes of Administration	Mechanism	Conclusion	Reference
IGF1	5 Gy	Roscovitine	25 mg/kg 100 mg/kg	Intraperitoneal injection	Female FVB mice	A cyclin-dependent kinase inhibitor that acts to transiently inhibit cell cycle progression and allow for DNA repair in damaged tissues.	Induction of transient G2/M cell cycle arrest by roscovitine allows for the suppression of apoptosis, thus preserving normal salivary function following targeted head and neck irradiation.	[123]
	5 Gy	IGF1	5 μg/mouse	Tail-vein injection	FVB, C57BL/6 J, and *Prkcz−/−* mice	Administration of IGF1 post-radiation maintains the activation of aPKCζ and partially rescues Yap’s cellular localization in label-retaining cells while restoring salivary function.	aPKCζ is required to restore the function of irradiated SGs using IGF1. This restoration process involves the maintenance of aPKCζ phosphorylation and the modulation of nuclear translocation of Yap in acinar LRCs in an aPKCζ-dependent fashion.	[125]
	2 or 5 Gy	IGF1	10 ng/mL	Intravenous injection	Human UMSCC1, UMSCC23, CAL 27, A-253, and FaDU cells	Not stated	Head and neck squamous carcinoma cell xenografts treated with concurrent radiation and IGF1 also exhibit significant tumor growth delay.	[124]
	5 Gy	IGF1	5 μg	Vehicle injection	Female FVB mice	Not stated	Post-therapeutic IGF1 treatment restores SG function, potentially through the normalization of cell proliferation and the improved expression of amylase.	[16]
	5 Gy	Intravenous recombinant human IGF1	5 μg	Intravenous	FVB females	In parotid glands of irradiated mice pretreated with IGF1, reduced ΔNp63 protein facilitates a p53-mediated increase in p21 expression that leads to G2/M arrest.	Radiation-induced increases in ΔNp63 protein correspond with enhanced binding to the p21 promoter and decreased p21 transcription in irradiated parotid glands after 8 h compared to parotid glands pretreated with IGF1.	[122]
Targeted therapy	15 Gy	HBOT	Once a day for five consecutive days a week	-	Female C3H mice	Not stated	HBOT can inhibit the TGFβ-pathway in irradiated SGs and restrain consequential radiation-induced tissue injury.	[127]
	15 Gy	SIM	10 mg/kg	IP injection	Male ICR mice	The protective benefits of SIM may be attributed to scavenging malondialdehyde, remitting collagen deposition, and reducing and delaying the elevation of TGFβ1 expression induced by radiation.	SIM remitted the reduction of saliva secretion and restored salivary amylase activity.	[126]
	10 or 15 Gy	Imatinib dasatinib	Imatinib (50 mg/kg) and dasatinib (20 mg/kg)	Not stated	The ParC5 cell line HNSCC cell lines	PKCδ is required for IR-induced apoptosis in the SG and that blocking activation of PKCδ with TKIs suppresses apoptosis.	Dasatinib and imatinib provide the profound and durable protection of SG function in vivo when delivered in conjunction with a single or fractionated doses of IR.	[128]
	Not stated	Imatinib dasatinib	Dasatinib (20 mg/kg)	Not stated	ParC5 cell line 293T cells	TKIs effective against c-Src and c-Abl are able to block multiple key regulatory steps necessary for PKCδ nuclear localization, leading to suppression of apoptosis both in vitro and in vivo.	TKIs is useful for the protection of nontumor tissues in patients undergoing radiotherapy of the head and neck.	[63]

IGF1, insulin-like growth factor-1; PKCζ, protein kinase C ζ; HBOT, hyperbaric oxygen therapy; TGF-β, transforming growth factor-β; SIM, simvastatin; IP, intraperitoneal; ICR, Institute of Cancer Research; HNSCC, head and neck squamous cell carcinoma; PKCδ, protein kinase C-delta; IR, irradiation; TKI, tyrosine kinase inhibitor; and SG, salivary gland.

**Table 2:** Stem cell treatment of radiation-induced SG injury.

Study	Cells	Radiation Dose	Treatment	Detection Method	Pathway to Restore SG Function	Treatment Effect	Conclusion	Reference
Martti Maimets et al.	SG ductal EpCAM and cells	15 Gy	-	SFR	Wnt signals	Nuclear β-catenin.	Stimulating self-renewal and long-term expansion of SG organoids, containing all differentiated SG cell types.	[136]
Bo Hai et al.	Mouse SGs and cultured human salivary epithelial cells	15 Gy	Sonic hedgehog (Shh) transgene or Smoothened Agonist in mouse salivary glands, adenovirus encoding Gli1 in human salivary epithelial cells	Detection of Ptch1-lacZ reporter gene and endogenous Hedgehog target gene expression	Transient activation of Hedgehog pathway	Preservation of salivary stem/progenitor cells, the Bmi1 signaling pathway, parasympathetic innervation, Chrm1/HB-EGF signaling, and expression of neurotrophic factors after IR by transient Hh activation.	Transient Shh overexpression activated the Hedgehog pathway in ductal epithelia, thus rescuing salivary function in male mice, which was related to the preservation of functional SSPCs and parasympathetic innervation.	[131]
Yoshinori Sumita et al.	Salivary epithelial cells	18 Gy	BMDCs	The expression of stem cell markers (Sca-1 or c-kit)	Direct differentiation of donor BMDCs into salivary epithelial cells	An increased ratio of acinar-cell area and approximately 9% of Y-chromosome-positive (donor-derived) salivary epithelial cells in BMDC-treated mice.	A cell-therapy approach, the transplantation of BMDCs via intravenous injections, can regenerate radiation-damaged tissue and rescue SG functions.	[150]
Xiaohong Peng et al.	SG sphere derived cells of Gdnf hypermorphic (Gdnfwt/hyper) and wild type mice (Gdnfwt/wt)	0, 1, 2, 4, and 8 Gy	-	QPCR and immunofluorescence	GDNF–RET signaling pathway	MSGSC of Gdnfwt/hyper mice showed high sphere-forming efficiency upon replating.	GDNF does not protect mSGSCs against irradiation but seems to promote mSGSC proliferation through the GDNF–RET signaling pathway.	[134]
Julie P Saiki et al.	Adult WT and *Aldh3a1−/−* murine SMGs	15 and 30 Gy	D-limonene	PAS staining annexin V PI	ALDH3A1 plays an important role in protecting SSPCs from IR-induced injury by increasing aldehyde scavenging.	ALDH3A1 activation with d-Limonene reduces aldehydic load, improves sphere growth, and reduces apoptosis in SMGs.	d-limonene may be a good clinical candidate for mitigating xerostomia in patients with head and neck cancer receiving radiation therapy.	[145]
Nan Xiao et al.	C57BL/6 mice C57BL/6-Tg(UBC-GFP)30Scha/J mice	15 Gy	Lin–CD24+ c-Kit+Sca1+ stem cells (GDNF)	PAS staining revealed more functional and intact acini in GDNF-treated SMGs than in saline glands	Not stated	Administration of GDNF improved saliva production and enriched the number of functional acini in submandibular glands of irradiated animals, as well as enhancing salisphere formation in cultured salivary stem cells, but it did not accelerate growth of head and neck cancer cells.	GDNF pathway may have potential therapeutic benefit for the management of radiation-induced xerostomia.	[135]
Bo Hai et al.	C57BL/6 mice	15 Gy	Adenoviral vector encoding GFP or rat Shh	Detection of SA-β-gal activity	mRNA levels of Chek1, Egfr, and survivin were significantly upregulated by the transfer of Shh inhibition of GDF15 upregulation	Shh gene transfer represses IR-induced cellular senescence by promoting DNA repair, decreasing oxidative stress (which is mediated through upregulating expression of genes related to DNA repair such as survivin and miR-21), and repressing expression of the pro-senescence gene Gdf15 likely downstream of miR-21.	Repressing cellular senescence contributes to the rescue of IR-induced hyposalivation by the transient activation of Hh signaling, which is related to enhanced DNA repair and decreased oxidative stress in SMGs.	[7]
Junye Zhang et al.	C57BL/6 mice	18 Gy	DFO	SFR	DFO improved angiogenesis via activating HIF–1α–VEGF Pathway	In addition to the restoration of salivary function, DFO administration also increased angiogenesis and the number of stem/progenitor cells while reducing the apoptosis of acinar cells.	DFO could prevent the radiation-induced dysfunction of SGs in mice.	[133]

SG, salivary gland; BMDCs, bone marrow-derived cells; Shh, Sonic hedgehog; IR, irradiation; DFO, deferoxamine; SSPC, salivary stem/progenitor cells; BMDCs, bone marrow-derived cells; GDNF, glial cell-derived neurotrophic factor; mSGSCs, mouse SG stem cells; ALDH3A1, aldehyde dehydrogenase 3A1; SMGs, submandibular glands; PAS, periodic acid Schiff; PI, propidium iodide; GFP, green fluorescent protein; and SFR, salivary flow rate.

**Table 3:** Gene transfer treatment of radiation-induced SG injury.

Scheme	Radiation Dose	Vector	Gene	Model	Detection Method	Pathway	Treatment Effect	Conclusion	Reference
Runtao Gao et al.	20 Gy	Serotype 2 and AAV2 vector	hAQP1 cDNA	Salivary hypofunction in minipigs	SFR	Not stated	In glands treated with the AAV2hAQP1 vector, a steady increase in parotid SFR was seen, such that by week 8, they were on average 35% of pre-IR values—nearly 1 mL/10 min.	The AAV2hAQP1 vector could be useful for targeting IR-surviving duct cells in previously irradiated head and neck cancer patients and providing them with a more extended (months) means of increasing salivary flow versus the AdhAQP1 vector (days).	[151]
C Zheng et al.	Not stated	hCMVp	hAQP1 cDNA	Female C3H mice and male Wistar rats	PCR assays Measurement of cell volume	Not stated	Not stated	The hCMVp in AdhAQP1was probably not methylated in transduced human SG cells of responding subjects, resulting in an unexpectedly longer functional expression of hAQP1.	[154]
L Guo et al.	7.5 or 9 Gy	Hybrid serotype 5 adenoviral vector	FGF2 cDNA	Parotid glands of minipigs	Local blood flow rate measurement	Not stated	Compared to the IR and AdLacZ and IR groups, the salivary flow rates of the AdLTR2EF1α-FGF2 and IR group were significantly higher (*p* < 0.001) and only slightly changed from that of naive animals.	Pre-administration of AdLTR2EF1α-FGF2 prevented an IR-induced reduction in MVD in minipig parotid glands within 24 h after IR. Pre-administration of AdLTR2EF1α-FGF2 also led to significantly higher levels of salivary secretion than those seen with untreated but irradiated minipigs and with minipigs that were irradiated and administered a control vector.	[118]
Chang yu Zheng et al.	15 Gy	AdLTR2EF1α-hKGF	KGF	Female C3H mice	Salivary flow	Not stated	The SFR from 2 groups (Mice receiving 15 Gy and AdControl) were also significantly lower than those of irradiated mice treated with AdLTR2EF1α-hKGF (*p* < 0.001). In contrast, the salivary flow rates from the no-IR and AdLTR2EF1α-hKGF plus IR groups were not significantly different (*p* = 0.065).	The hKGF gene transfer had no effect on the growth or radiation sensitivity of a model SCC. Transfer of the hKGF gene to SGs prior to both fractionated and single-dose IR substantially prevents salivary hypofunction.	[119]
Bo Hai et al.	15 Gy	Adenoviral vector	*Shh* delivery	B6;129-Ptch1tm1Mps/J (Ptch1-lacZ) mice and wild-type C57BL/6 mice	X-Gal staining and qRT-PCR analysis saliva flow rate	Hedgehog/Gli	Shh gene transfer is a feasible approach to restore SG function after radiotherapy, which functions through ameliorations of IR damage to the microvasculature and parasympathetic innervation by the upregulation of paracrine factors.	Transient activation of the Hedgehog pathway by gene delivery is promising to rescue salivary function after irradiation in both sexes.	[159]
Liang Hu et al.	20 Gy	Adenoviral vector	GFP or Shh	Healthy littermate BA–MA male miniature pigs	IHC qRT-PCR analysis	Hedgehog/Gli	Shh gene delivery 4 weeks after irradiation significantly improved stimulated saliva secretion and local blood supply up to 20 weeks; preserved saliva-producing acinar cells, parasympathetic innervation and microvessels as found in mouse models; and activated autophagy and inhibited fibrogenesis in irradiated glands.	The translational potential of transient activation of the Hedgehog pathway to preserve salivary function following irradiation.	[155]
Bo Hai et al.	15 Gy	Adenoviral vector	human Gli1 or GFP	*Ptch1-lacZ* mice	qRT-PCR analysis and X-gal staining	Hedgehog/Gli	Transient Shh overexpression activated the Hedgehog pathway in ductal epithelia; and this, after irradiation, rescued salivary function in male mice, which was related to the preservation of functional SSPCs and parasympathetic innervation.	Transient activation of the Hedgehog pathway has the potential to restore irradiation-induced SG dysfunction.	[131]
Bo Hai et al.	Not stated	Serotype 2 AAV2 vectors	hEpo	Miniature pig	Enzyme-linked immunosorbent assay	-	AAV2 vectors mediate extended gene transfer to miniature pig parotid glands and should be useful for testing pre-clinical gene therapy strategies aiming to correct SG radiation damage.	AAV2 vectors mediate extended gene transfer to miniature pig parotid glands and should be useful for testing pre-clinical gene therapy strategies aiming to correct SG radiation damage.	[163]

SFR, salivary flow rates; AAV, adeno-associated virus; AQP1, aquaporin 1; IR, irradiation; hEpo, human erythropoietin; qPCR, quantitative PCR; Shh, sonic hedgehog; SSPC, salivary stem/progenitor cells; GFP, green fluorescence protein; IHC, immunohistochemistry; KGF, keratinocyte growth factor; FGF2, fibroblast growth factor-2; MVD, microvascular density; hCMVp, human cytomegalovirus enhancer/promoter; and SG, salivary gland.
